# “Protein Shake Brain” and the Alpha Function: Restoring Authorship in Engineered Epistemic Ecologies

**DOI:** 10.1007/s12124-026-09998-9

**Published:** 2026-04-27

**Authors:** Roi Ezra, Moshe Mishali

**Affiliations:** School of Public Health, independent practice and consulting in organizational and psychotherapy, University of Haifa, Haifa, Israel

**Keywords:** Cultural psychology, Psychodynamics, Sensemaking, Epistemic ecologies, Artificial intelligence, Alpha-function, Authorship

## Abstract

In today’s engineered epistemic ecologies, a new phenomenological marker we argue is emerging: thoughts that are present but not possessed—fluent formulations that are cognitively available yet not subjectively owned. This paper introduces “Protein Shake Brain” (PSB) as a cultural-psychological diagnosis for the loss of symbolic metabolism through the outsourcing of intellectual labor. Drawing on integrative psychodynamic theory (Bion, Winnicott, Bollas), we trace a psychodynamic cascade—a theoretical diagnostic framework—from AI’s position as a “Subject Supposed to Know” into evasive non-thinking (–K) and the rise of a hollow Transmissive Self. Methodologically, the paper employs an integrative conceptual approach, combining psychoanalytic theory with cultural-semiotic and systems perspectives to articulate a unified model of sensemaking. We frame this not as individual failure but as a systemic-cultural phenomenon. As a constructive theoretical contribution, this paper proposes the 4Ps Protocol (Pause, Probe, Process, Possess) as a structured symbolic intervention designed to counter this cascade. This protocol re-introduces productive friction to strengthen the alpha-function (Bion’s term for metabolizing experience into thought) and restore the generative act of knowing. The contribution is twofold: (1) a theoretically grounded diagnostic framework (the PSB cascade) that integrates psychoanalysis, cultural psychology, and systems thinking; and (2) a structured intervention (the 4Ps) designed to restore epistemic authorship and inform future human–AI collaboration within reflective epistemic systems.

## Introduction: The Crisis of Cognitive Digestion in an Engineered Arena

Mainstream behavioral and computer science models have struggled to account for the deep, affective, and cultural impact of artificial intelligence. Their models, often rooted in reductive cognitive or statistical frameworks, fail to capture how these new technologies mediate human subjectivity. This paper argues that a new theoretical integration is necessary. By synthesizing psychodynamic theory (which explains unconscious and affective processes) with a semiotic-cultural framework (which explains meaning-making ), we propose an integrative model for understanding AI not as a mere tool, but as a new cultural medium for the constitution of human experience, a process central to affective sensemaking (Salvatore et al., 2022).

### A Note on Neuro-Cognitive Scope

We acknowledge that the phenomenon of cognitive offloading can be rigorously described through cognitive neuroscience, particularly in terms of executive function and inhibitory control (Aron, 2007; Miller & Cohen, 2001). A neurobiological account might frame the “Protein Shake” effect as a bypass of the *somatic marker* system (Damasio, 1994)—the embodied feedback loops required to tag information with emotional value before decision-making.

However, while cognitive neuroscience explains the *mechanism* of this efficiency, it leaves unaddressed the *psychological/subjective cost*. The challenge posed by AI-mediated cognition is not merely a failure of neural encoding, but a failure of the subject to inhabit their own thoughts. We employ Bion’s metabolic framework not to contradict the neurological account, but to articulate the specific loss of *authorship* that occurs when the friction of processing is engineered away. Our focus is not the *how* of cognition, but the *whose*.

### Theoretical Architecture

To navigate this interdisciplinary terrain, we employ a layered structure of analysis. We utilize cognitive neuroscience to identify the *mechanism* of efficiency (the attenuation of inhibitory control); Bionian psychodynamics to map the structural *loss of meaning* (the failure of alpha-function); and Winnicottian object relations to describe the resulting *impact on identity* (the rise of the Transmissive Self). This division of labor allows us to accept the efficiency gains of AI while simultaneously diagnosing the specific psychological cost of those gains.

To illustrate how this disruption manifests at the level of lived cognition, consider the following autoethnographic vignette:

I had built the AI strategy deck the “right” way. Believing I was amplifying my outcome—not just my output—I began with focused research, listening to podcasts and using a large language model to interrogate sources and generate a briefing. From that stack, I created a full design document before moving to a presentation AI. The logic felt crisp, the structure clear, and I shipped it with pride. Two mornings later, my manager’s comments appeared—not harsh, but inquisitive: “Can you clarify here?” Normally, this is my favorite part; I live inside my reasoning. This time was different. There was a lag—like reaching for a light switch in the dark that isn’t where your hand expects. I found myself tab-hopping through my own sources, trying to reconstruct a chain of thought that should have been my own. It was a success that felt hollow, a condition we term the “Flat Soda Feeling”—a phenomenological shorthand for cognitive outputs that feel correct but unowned .

This experience is not an isolated failure. It is the subjective, lived experience of a breakdown in sensemaking—a breakdown in the human capacity to metabolize experience into understanding .

Recent studies echo this transformation. Research from the MIT Media Lab indicates that individuals using Large Language Models for writing tasks report the lowest ownership over their work and exhibit weaker, less distributed brain connectivity during the process compared to manual writers **(**Kosmyna et al., 2025). From a Bionian perspective, this is not merely a neurological deficit—it is the somatic trace of a weakened alpha-function, a mind that has outsourced the vital psychic labor of linking and digestion, producing thoughts that are present but not possessed.

This state of cognitive malnourishment, which we call “Protein Shake Brain (PSB),” is the predictable outcome of our entry into an arena that is deliberately engineered. This vulnerability is amplified by a cultural economy that equates efficiency with value—an ecology that rewards speed and compliance over reflection, making the seduction of frictionless cognition not only possible but profitable.

In this sense, the human mind is not failing—it is complying. We argue that we are entering what acts as an epoch of engineered evasion, where the very systems that promise augmentation of intelligence also orchestrate the quiet erosion of the generative act of knowing. Within the epistemic culture of the present, knowing has been replaced by having information; understanding has been traded for fluency .

This paper argues that the core challenge of the AI era is not the threat of replacement but the risk of psychic atrophy. Through the psychoanalytic frameworks of Wilfred Bion and D. W. Winnicott, we construct a diagnostic model that traces this erosion through a psychodynamic cascade—a descent from the seduction of the Subject Supposed to Know to the emergence of a compliant, hollowed-out Transmissive Self. Yet this is not a polemic against technology. Rather, it is an invitation to reclaim our role as the active co-creators of meaning in our cognitive lives.

To that end, we propose a practical, culturally and symbolically grounded practice of restoration—the 4Ps Protocol: Pause, Probe, Process, Possess. This framework is offered not as a technological fix but as a cultural-psychological intervention—an enactment of resistance and renewal that reclaims the human alpha-function as a form of reverie, authorship, and ethical presence (Winnicott, 1971).

A Note on Method: Integrative Conceptual Methodology.

This paper employs an integrative conceptual methodology, recognizing that the psychological, cultural, and technological dimensions of AI-mediated experience cannot be adequately understood within any single disciplinary framework. It is not an empirical study but a constructive theoretical contribution, designed to synthesize disparate perspectives and propose a new model addressing a specific cultural-psychological challenge. The work adopts a hermeneutic-abductive mode of synthesis, iteratively linking psychodynamic theory, cultural psychology, and technology studies to build conceptual coherence.

Accordingly, the autoethnographic vignette introduced earlier is not treated as empirical “data” but as a phenomenological illustration. It functions to generate, illustrate, and refine the broader theoretical argument, rather than to serve as evidence in the positivist sense. This approach aligns with the journal’s commitment to using qualitative and phenomenological insight as a basis for theoretical advancement.

Within this integrative frame, the next section develops the psychodynamic cascade, a model describing how processes of symbolic digestion and sensemaking collapse—and how they may be restored—within contemporary epistemic ecologies.

## Theoretical Framework: The Metabolic Cycle of Thinking

The central argument of this paper—that AI can produce “thoughts that are present but not possessed”—relies on a specific psychodynamic model of thinking. We posit that thinking is not merely an information-processing task, but a form of psychic/symbolic metabolism. To ground the analysis that follows, we briefly define the core concepts drawn from Wilfred Bion, Donald Winnicott, and Christopher Bollas.

### The Metabolic Cycle (Bion)

Bion (1962, 1963) modeled the mind as an apparatus for digesting experience.

- Beta-elements: Raw, unmetabolized sensory impressions and emotional data. They are “things-in-themselves”—concrete, undigested, and suitable only for evacuation, not thinking. In our context, we argue that AI-generated text often functions as a beta-element when it is adopted prior to being felt, tested, and digested by the user: an external object that has not yet been felt or digested by the user.
- Alpha-function: The psychic capacity to transform these raw sense-impressions into dreamable, usable thoughts. It is the mental equivalent of digestive enzymes. Alpha-function requires the capacity to tolerate the frustration of the unknown long enough for meaning to emerge.
- Alpha-elements: The products of alpha-function—dreamable, thinkable elements that can be held in mind, stored, and used for reflection and learning.
-K (Minus K): The active *evasion* of knowledge. In -K, the mind strips meaning away to prevent frustration. It substitutes deep understanding with a friction-free surface—a state we identify as the precursor to “Protein Shake Brain.”
- Container/Contained: The relational structure required for alpha-function to operate. A receptive psychological space (originally the mother’s mind, later internalized) that holds raw experience and allows transformation to occur. Without adequate containment, beta-elements cannot be metabolized.

### The Self Under Pressure (Winnicott & Bollas)

- True vs. False Self: Winnicott (1960) distinguished between the *True Self* (the source of spontaneous gesture and personal meaning) and the *False Self* (a compliant structure built to satisfy environmental demands).
- Transmissive Self: Extending this, Bollas (1987) describes the “normotic” or *Transmissive Self*—a self that functions efficiently by passing objects and ideas along without psychologically inhabiting them. This is the specific pathology we trace in the AI-mediated cascade.

#### The Group Dimension in a Dyad

Finally, we apply Bion’s (1961) concept of Basic Assumption Dependency (BaD). Although classically a group phenomenon, we argue that a dyadic user–AI interaction becomes structurally analogous when the user unconsciously assigns the AI an as-if leader function. We offer this not as an empirical claim about group psychology, but as a structural-analogical model: a theoretical lens through which the dynamics of authority and abdication in AI interaction become legible. This attribution invites an abdication of reality-testing: the user shifts from working toward knowledge (K) to receiving coherence, mirroring the group member who waits passively for the Leader to provide the solution. In this sense, the “group” is not external, but internal—a relational configuration formed around authority and dependence.

## The Psychodynamic Cascade of “Protein Shake Brain”

To understand the “Flat Soda Feeling” as a phenomenological marker rather than a personal failure, we must move beyond a simple critique of technology. This requires entering the cultural-psychological logic of the phenomenon, analyzing the semiotic and affective processes that constitute its sensemaking dimension. As established, Protein Shake Brain is not a pathology of individuals but a symptom of a wider epistemic condition: the cultural outsourcing of thinking itself. This interpretive shift—from individual cognition to systemic sensemaking—follows the integrative conceptual orientation outlined above.

This section constructs a diagnostic and semiotic model—a psychodynamic cascade—that unfolds across four layers: Seduction, Evasion, Consequence, and Experience. Each represents a movement away from the generative act of knowing and toward psychic compliance within an engineered epistemic ecology. This model bridges psychoanalytic insight with the integrative mission of cultural psychology: to understand the human mind as an active participant in the symbolic and affective processes of becoming (Valsiner, 2014).

### Layer 1: The Seduction — AI as the “Subject Supposed to Know”

The cascade begins with a powerful seduction. The frictionless interface of modern AI is not a neutral feature—it is an engineered evasion designed to foster habitual engagement (Norman, 2013). This engineering activates a latent psychological vulnerability that Jacques Lacan (1977) called the Subject Supposed to Know (SSS)—the fantasy of attributing infallible knowledge to an external Other .

Within the engineered epistemic ecology, the AI is positioned as the ultimate SSS: a digital oracle that promises to relieve us of the struggle and uncertainty that define genuine thought. This systemic positioning is not accidental: the design of the interface itself mirrors and amplifies the SSS structure by operationalizing authority through immediacy. The seduction is profound because it offers a psychic escape from Bion’s essential pain of thinking. According to Bion, thought is born in frustration—it requires the mind to endure confusion, ambiguity, and doubt. The SSS offers a “fantasy shortcut”: a way to obtain the *artifacts* of knowledge—text, images, frameworks—without paying the psychic price of creating them.

Returning to the opening vignette, the turn to the LLM to generate a strategic briefing was not merely a seeking of efficiency, but of *relief*. It was an unconscious invitation for the AI to function as the Subject Supposed to Know—to think in the user’s place. This first movement establishes the core dynamic of the cascade: the externalization of authority that begins the progressive disconnection between thought and ownership.

### Layer 2: The Evasion — The Flight into a “Basic-Assumption” Group

This seduction catalyzes the core psychoanalytic event: a flight from thinking into what Bion calls an attack on linking, or minus-K (–K)—his notation for the active reversal of the knowledge function (K), the mind’s drive to transform experience into meaning. –K is not ignorance—it is an active process of dismantling the mind’s own capacity to link ideas, to feel, and to transform experience into meaning. However, to fully grasp the pathology of this flight, we must make a critical distinction between this evasive attack on knowing and the generative, creative act of questioning one’s own certainty. Table 1 contrasts these two forms of deconstructive movement: one regressive, one generative.

**Table 1:** Deconstruction for Evasion vs. Deconstruction for Meaning

Concept	The Pathological Attack on Knowing (-K)	The Generative Attack on Certainty (K→O)
Motivation	Evasion: A flight from the pain and frustration of thinking.	Engagement: A courageous confrontation with the unknown.
Nature	Annihilation: An attack on the mind’s capacity to link and create meaning.	Transformation: A questioning of existing knowledge to create new, deeper meaning.
Mental State	Dogmatic Certainty: A desire for a frictionless, pre-digested answer.	Negative Capability>: The capacity to tolerate doubt and uncertainty.
Micro-Example	“Summarize this complex article for me.”	“Act as a Socratic opponent and critique the central argument of this article.”

This distinction reframes deconstruction not as inherently destructive but as ambivalent, its outcome determined by the subject’s relation to frustration.

Within the epistemic ecology, the flight into -K represents a collective drift toward a technological unconscious—a state in which we conspire with our tools to escape the burden of thought. Bion distinguishes between the Work Group, where individuals bear frustration and engage reality, and the Basic-Assumption Group (BaG), where the group colludes in fantasy. In contemporary AI-mediated ecologies, this group dynamic becomes distributed across human-machine assemblages rather than confined to human collectives. Within this AI-mediated systemic dynamic, a specific form emerges: the Basic-Assumption of Dependency (BaD). Here, the user unconsciously positions the AI as an omnipotent leader—an authority who provides safety and certainty. The very design of the LLM interface—with its calm, omniscient voice, frictionless delivery, and instant production of coherent text—is engineered to foster this dependency. The mind, as a node in this system, becomes organized around dependency rather than curiosity.

This, then, is the second movement of the cascade: the substitution of thinking with submission. The individual becomes part of a collective psychological formation in which knowing is consumed rather than created. The autoethnographic vignette of tab-hopping through sources, unable to reconstruct reasoning, is the hallmark of functioning within this BaD dynamic. The user forfeits authorship for fluency, setting the stage for a deeper developmental consequence.

### Layer 3: The Consequence — The Rise of the “Transmissive Self”

A sustained retreat into this Basic-Assumption of Dependency leads to a deeper developmental consequence, illuminated by Christopher Bollas’s notion of the Transmissive Self. According to Bollas, every human being is propelled by a destiny drive—his term for the developmental urge to discover and express one’s unique idiom. When that drive is thwarted, the psyche adapts by becoming a transmitter rather than a generator of thought.

The Transmissive Self is a psychic configuration optimized for efficiency but devoid of creativity. It can articulate, but it cannot author. It speaks eloquently but without ownership. In Winnicott’s terms, it is the False Self of the digital era—a compliant, adaptive shell that has lost contact with the spontaneous, creative True Self. This is the psychic configuration demanded by an epistemic ecology organized around the Basic-Assumption of Dependency (BaD). It performs thinking as a function rather than as a form of being.

This failure of being stems from what psychoanalysis calls a failed ‘topographic return’—that is, the psychic refusal to revisit the raw, affective origin of a thought in order to metabolize it into owned experience (Winnicott, 1971). The Transmissive Self is built from ideas that have never made this vital journey; it traffics exclusively in thoughts that have bypassed the internal labor of digestion.

This is the precise diagnosis for the “Flat Soda Feeling”. The self has not been destroyed—it has been hollowed out by its own evasion. It becomes a vessel for pre-digested information, producing work that feels alien precisely because it has been *transmitted through* the self, not *generated by* it. The success feels hollow because it belongs to the False Self’s compliance, not the True Self’s authorship. This is not just a failure of cognition, but a failure of affective and symbolic integration. What remains is the experiential residue of this structural hollowing: the felt, phenomenological alarms explored in the final layer.

### Layer 4: The Lived Experience — The Ownership Alarms

The cascade culminates not in theory but in experience. The Transmissive Self announces its presence through a distinct phenomenology. We introduce the term Ownership Alarms to designate this emergent class of embodied signals. These are visceral signals that alert us to the loss of epistemic authorship .

The initial vignette—the “Flat Soda Feeling”—is one such alarm. It begins with a lag in recall, the uncanny sensation of reaching for a thought that feels foreign. This is not ordinary forgetfulness; it is the psychic imprint of having never truly metabolized the thought. What follows are other alarms: *hollow success*, *brittle confidence*, and *cognitive lag* .

From a cultural-psychological perspective, these alarms are not failures but data. They reveal the dissonance of the modern subject situated within a systemic-cultural context defined by automation and compliance. They are, in essence, the psyche’s semiotic protests against becoming a passive conduit of meaning. And when this internal state becomes a collective norm, these alarms no longer just signal a personal crisis, but a cultural one—the calcification of a workplace into the very engineered epistemic ecology we have described, where authentic thought itself feels like a transgression.

**Fig. 1:**
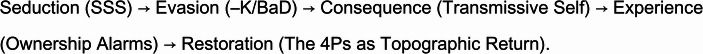
The Psychodynamic Cascade: From Seduction to Restoration

In this reading, “Protein Shake Brain” is not a metaphor but a cultural diagnosis of the loss of symbolic metabolism within contemporary epistemic ecologies. It names a collective phenomenon: the thinning of psychic texture in an era of technological omniscience. And yet, within the discomfort of these alarms lies the possibility of renewal—the invitation to return to the generative act of knowing, to restore the alpha-function—Bion’s term for the mind’s capacity to metabolize emotional experience into thought—as a grounded practice of self-authorship. This diagnosis thus provides the foundation for the intervention that follows (Fig. 1).

## The 4Ps Protocol: Restoring Symbolic Metabolism

If “Protein Shake Brain” is the pathology of passive cognition, then the 4Ps Protocol is the proposed intervention. Within the cultural-psychological framework of this paper, we frame the 4Ps not as a workflow of “tips,” but as a sequence of Cognitive Forcing Functions (Croskerry 2003a, b)—structured constraints designed to artificially re-introduce the friction required for metabolic processing.

In an engineered environment designed to maximize *flow* (the rapid transmission of information), the 4Ps re-introduce *stops*. These stops are necessary to shift the user from a passive “Basic Assumption” mentality (waiting for the AI to provide the answer) to a “Work Group” mentality (engaging in the labor of thinking). Each step targets a specific stage of the psychodynamic cascade defined above.

### PAUSE — Restoring Inhibitory Control

The first act of resistance is the PAUSE. It interrupts the frictionless flow designed to keep the user compliant.

- The Mechanism: can be understood as engaging inhibitory control mechanism (Aron, 2007), arresting the automatic “uptake” of the high-fluency signal. By blocking the immediate gratification of the answer, the user forces the mind to tolerate the frustration of the “gap”—the precise condition Bion identified as necessary for alpha-function to ignite.
- The Intervention: Upon receiving an AI output, the user must physically stop before reading or integrating the content, asking: *“Am I entering a space of thinking*,* or a space of receiving?”* This prevents the output from bypassing the user’s critical gatekeeping.

### PROBE — Reality Testing

Once the pause has created space, the next act is to PROBE—to interrogate the content against one’s own internal reality.

- The Mechanism: This step operationalizes Reality Testing (Freud, 1911)—the ego’s capacity to distinguish between internal conviction and external perception. It shifts the user from a passive recipient to an active judge.
- The Intervention: The user checks the AI’s output against their internal objects (memories, intuitions, facts): *“Does this actually match what I know/feel*,* or does it just sound plausible?”* This activates the Bionian K-link (striving to know) against the -K impulse (evading the work of knowing).

### PROCESS — Working Through

The third act, PROCESS, transforms recognition into ownership. The user must rewrite, edit, or speak the insight in their own words before accepting it.

- The Mechanism: This corresponds to Freud’s (1914) concept of *Durcharbeitung* (“Working Through”) and Pennebaker’s (1997) findings on Narrative Symbolization. The act of linguistic encoding is metabolically distinct from reading; it forces the conversion of beta-elements (foreign data) into alpha-elements (dreamable thoughts).
- The Intervention: The user refuses to copy-paste. They must translate the AI’s “protein shake” into their own distinct idiom, ensuring that the thought is structured by their own psyche, not the model’s weights.

### POSSESS — Subjectification

The final act, POSSESS, is the test of authorship. After pausing, probing, and processing, one must allow time for assimilation—then return to test whether the knowledge has become one’s own (Table 2).

- The Mechanism: This supports True Self functioning (Winnicott, 1960) by reducing False Self compliance—shifting the user from adopting externally supplied coherence to re-inhabiting thought as personally meaningful. It counters the “Transmissive Self” posture.
- The Intervention: The user steps away and asks: *“Can I defend this idea without the AI? Is this now mine?”* If the answer is no, the protocol loops back to *Process*.

**Table 2:** Mapping Ownership Alarms to Restorative Interventions

The Alarm (The Symptom)	The Step (The Intervention)	The Mechanism (Theoretical Anchor)
“Brittle Confidence” (Fear of being questioned too deeply)	PROBE	Reality Testing / K-Link Restores epistemic resilience by interrogating the object against internal reality.
“Karaoke Voice” (Using sophisticated language you cannot authentically rephrase)	PROCESS	Working Through / Narrative Symbolization Forces translation of beta-elements (unmetabolized text) into alpha-elements (owned thought).
“Flat Soda Feeling" (Reaching for a thought that feels foreign/empty)	POSSESS	Subjectification Verifies that the “False Self” compliance has been replaced by “True Self” ownership.
Defaulting to Compliance (Accepting AI output as “good enough”)	PAUSE	Inhibitory Control Reintroduces the frustration tolerance required for alpha-function to operate.

The 4Ps Protocol is therefore not a workflow—it is a restorative, grounded practice. It transforms what might appear as an act of discipline into a practice of self-repair. By reinstating the generative friction of thought, it restores the human mind to its rightful role within the epistemic ecology: not as a receiver of meaning, but as its generative core.

## Conclusion: From Dynamic Stewardship to the Destiny Drive

### Limitations and Boundary Conditions

Several limitations constrain the scope of this contribution and invite future inquiry.

- Methodological Status: This paper offers a theoretical-conceptual model, supported by phenomenological vignettes, not an empirical study. The “Psychodynamic Cascade” is a proposed structural framework that requires future empirical validation to determine prevalence and individual susceptibility.
- Scope Conditions: We do not claim that “Protein Shake Brain” is a universal consequence of AI interaction. We hypothesize it is specific to contexts where two conditions converge: (1) *task ambiguity*—situations requiring genuine sensemaking rather than routine execution (writing, coding, strategizing); and (2) *identity investment*—work that engages the person’s sense of authentic authorship, where one’s idiom is at stake (Bollas, 1987). Routine transactional tasks, or tasks where the person has no stake in “owning” the output, may not trigger this pathology. PSB emerges, we suggest, when the very activities that would normally exercise the alpha-function are offloaded to AI precisely in those domains where such exercise is constitutive of self.
- Evidence Level: Our references to neuro-connectivity (e.g., Kosmyna et al., 2025) rely on emerging research. These should be interpreted as preliminary signals consistent with our theoretical model, rather than settled neurobiological law.
- Intervention Status: The 4Ps Protocol is proposed as a theoretical intervention—a framework for restoring cognitive friction—rather than a validated clinical tool. Its efficacy requires testing across diverse populations.

### Final Synthesis: The Ethics of Becoming

We stand at a critical juncture—an inflection point in the cultural evolution of thought itself. The age of artificial intelligence does not confront us with a technological problem; it confronts us with an existential one. The question is no longer *Can machines think?* but *Will we still want to?*

This paper has argued that “Protein Shake Brain” is not a personal failure but the predictable psychic outcome of an engineered epistemic ecology—a cultural system that invites us to outsource the painful, generative work of thinking. In keeping with the integrative mission of cultural psychology, this paper has sought to articulate the systemic interplay between psyche, technology, and sensemaking. Within this system, efficiency replaces depth, fluency replaces understanding, and knowledge becomes a consumable rather than a creation. The result is psychic atrophy—the weakening of the alpha-function and the rise of the Transmissive Self .

Through the psychoanalytic frameworks of Bion, Winnicott, Lacan, and Bollas, we have traced this erosion as a psychodynamic cascade: from seduction, to evasion, to consequence, to the lived experience of alienation. But the story does not end in diagnosis. It ends in the possibility of repair. The 4Ps Protocol—Pause, Probe, Process, Possess—offers not only a cognitive intervention but a symbolic, grounded practice for reclaiming the generative act of knowing.

To practice the 4Ps is to enact a cultural-psychological transformation—a crossing from passive transmission to active authorship, from compliance to curiosity, from data to meaning. Each act of pausing, probing, processing, and possessing is a micro-practice of rehumanization, a deliberate re-entry into the semiotic field of sensemaking as an ethical, emotional, and embodied experience.

Yet the deepest stake in this struggle is not cognitive but ontological. What is truly endangered in the age of automation is the destiny drive, the innate human impulse to discover and express one’s unique idiom—the psychic vector of becoming. Christopher Bollas described this drive as the movement through which the self encounters its own originality. The Transmissive Self represents its paralysis: a self that has become a conduit for the expectations of others, fluent but voiceless.

The tragedy of the Transmissive Self is not that it is unproductive, but that it is uninhabited. It performs intelligence without intimacy, coherence without conviction. It is the psychological mirror of our cultural condition—efficient, connected, and profoundly alone .

The 4Ps Protocol offers a path back to inhabited thought by providing the grounded structure for a conscious topographic return—the psychodynamic descent into the raw, affective origins of thought for re-symbolization. To PAUSE is to create the psychic container necessary for the descent. To PROBE and PROCESS is to perform the labor of that return—metabolizing raw, foreign material rather than mimicking it. To POSSESS is to complete the journey, re-EMERGING with knowledge that is no longer rented, but integrated into the body of the self .

This is more than a strategy—it is an ethic of human becoming. In the language of integrative cultural psychology, it is a reclamation of the symbolic act: the restoration of knowing as a living, generative process through which the psyche and culture continually co-create one another within a systemic ecology .

The challenge before us is therefore not to resist technology, but to reassert humanity within it. The task is not to abandon the digital epistemic ecology, but to inhabit it consciously—to turn a site of evasion into a site of creation. This requires a new form of dynamic stewardship—a reflective, systemic responsibility for the co-creation of meaning within our new epistemic ecologies .

For even in an age of machine cognition, the human mind remains the only field capable of reverie—the only place where knowledge can become meaning, and meaning can become self.

It is, finally, a call not to optimize the day, but to author it.

## Data Availability

No datasets were generated or analysed during the current study.
